# Adding layers: Environmental and internal modulation of regional skin barrier functions

**DOI:** 10.1111/jdv.20734

**Published:** 2025-05-20

**Authors:** Joachim W. Fluhr, Razvigor Darlenski

**Affiliations:** ^1^ Institute of Allergology (IFA) Charité – Universitätsmedizin Berlin Berlin Germany; ^2^ Allergology and Immunology Fraunhofer Institute for Translational Medicine and Pharmacology ITMP Berlin Germany; ^3^ Department of Dermatology and Venereology Medical Faculty, Trakia University Stara Zagora Bulgaria; ^4^ Department of Dermatology and Venereology Acibadem City Clinic Tokuda Hospital Sofia Bulgaria

The review by Dajnoki et al.^1^ offers a comprehensive synthesis of current insights into the topographical variability of skin barrier functions. The authors emphasize the complex interplay among chemical, microbial, physical and immunological barriers and explain how these interactions contribute to region‐specific skin vulnerability and disease patterns. To complement and extend the discussion, we propose five key dimensions that could be further integrated into the concept of skin barrier variability.

Environmental conditions such as UV radiation, temperature, humidity, ozone, fine dust and airborne allergens (e.g. pollen) affect skin barrier integrity.^2^ Recent studies confirm that pollution, particularly ozone and pollen exposure, can induce barrier impairment.^3^ These factors vary by region, climate and season and directly interact with the skin's surface.

Lifestyle factors—such as engagement in sports, occupational exposure, cultural practices in hygiene and clothing and repetitive mechanical friction—further modulate the barrier. Psychological stress has also been shown to disrupt barrier homeostasis via neuroendocrine pathways.^4^

The neuronal (sensory) barrier should be considered as active player in barrier function. Sensory nerve fibres influence not only perception but also barrier function, regeneration, inflammation and immune response. Their role is especially relevant in conditions such as sensitive skin and atopic dermatitis.^5^

The ‘inside‐out’ modulation emphasizes the importance of internal factors in maintaining barrier homeostasis.^6^ Systemic hydration, nutrition, metabolism and hormonal regulation shape skin reactions to external exposures. Understanding skin function as part of a whole‐body network opens new perspectives for clinical intervention.

Anatomical regions differ functionally and in terms of microenvironmental conditions.^7^ Corneodesmosomes, vital for the cohesion of the upper stratum corneum, vary in stability and distribution. Occluded areas such as the axillae, groin, anogenital region, submammary folds and plantar surfaces experience elevated moisture, reduced ventilation and altered pH.^8^ These conditions affect microbiome composition and predispose individuals to infections and maceration.

Hormonal regulation, especially by sex hormones, affects sebaceous gland activity and modifies barrier characteristics in specific regions. Fluctuations during puberty, menstrual cycles and endocrine disorders all contribute to localized barrier behaviour.

In parallel, bacterial adhesion and microbiome diversity vary by site, influencing the establishment and function of local microbial communities. These interactions are key to understanding region‐specific disease patterns.

Building on the foundation laid by Dajnoki et al.,^1^ incorporating environmental, neuronal, systemic, lifestyle and regional factors will enrich our understanding of skin barrier dynamics and support the development of targeted site‐adapted dermatological strategies (Figure 1).

**FIGURE 1:**
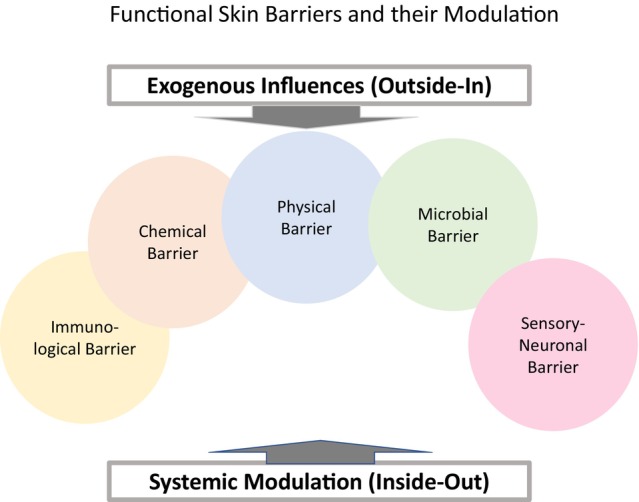
Expanded model of regional skin barrier modulation. The skin barrier comprises five interdependent functional layers: physical, chemical, microbial, immunological and sensory‐neuronal barriers. These layers maintain homeostasis and protection but are dynamically modulated by external (exogenous) influences such as environmental and lifestyle factors (outside‐in) and by internal (systemic) modulation, including hormonal, metabolic and neuronal factors (inside‐out). Regional anatomical differences (e.g. occlusion, gland density) and microbiome variation further shape site‐specific barrier properties and disease susceptibility.

## CONFLICT OF INTEREST STATEMENT

JWF and RD have no conflict of interest to declare related to this article.

## Data Availability

Data sharing not applicable to this article as no datasets were generated or analysed during the current study.
